# Gene Expression Profiles Associated with Molecular Subtypes and Pathological Response to Neoadjuvant Treatment in Surgical Breast Cancer

**DOI:** 10.3390/ijms27062564

**Published:** 2026-03-11

**Authors:** Sonia Baulies, Miguel Angel Molina-Vila, Francesc Tresserra, Ignacio Rodríguez, Yannick Hurni, Ana Giménez-Capitán, Silvia Cabrera, Rafael Fábregas

**Affiliations:** 1 Gynecologic Oncology and Breast Pathology Unit, Department of Obstetrics, Gynecology, and Reproduction, Dexeus Mujer, Dexeus University Hospital, 08028 Barcelona, Spain; 2 Pangaea Oncology, Laboratory of Molecular Biology, Coyote Research Group, Dexeus University Hospital, 08028 Barcelona, Spain; 3 Pathology Unit, Department of Obstetrics, Gynecology, and Reproduction, Dexeus Mujer, Dexeus University Hospital, 08028 Barcelona, Spain; 4 Statistics and Epidemiology Unit, Department of Obstetrics, Gynecology, and Reproduction, Dexeus Mujer, Dexeus University Hospital, 08028 Barcelona, Spain; 5 Department of Paediatrics, Obstetrics, Gynaecology and Preventive Medicine and Public Health, Faculty of Medicine, Universidad Autonoma Barcelona, 08193 Barcelona, Spain

**Keywords:** breast cancer subtypes, gene expression profiling, neoadjuvant chemotherapy, pathological response, predictive molecular markers

## Abstract

Chemotherapy has significantly improved survival in breast cancer and, in the neoadjuvant setting, contributes to tumor downstaging and increased rates of breast-conserving surgery while enabling in vivo assessment of tumor biology and chemosensitivity. Pathological complete response (pCR) is a key endpoint associated with favorable outcomes; however, tumor heterogeneity highlights the need for reliable predictive biomarkers. This study evaluated the mRNA expression of 13 candidate genes in relation to molecular subtypes and pathological response to neoadjuvant chemotherapy (NAC) to identify potential predictive and prognostic markers. Pretreatment core biopsies from 92 patients receiving NAC were analyzed by quantitative RT-PCR. Molecular subtypes were determined by immunohistochemistry (ER, PR, HER2, Ki67), and pathological response was classified using the Miller–Payne scale as good (MP 4/5) or poor (MP 1–3). Multivariate logistic regression assessed associations between gene expression, subtype, and pCR. Hormone receptor-positive tumors showed significantly higher expression of *AXL*, *FGFR1*, *RAP80*, *GAS6*, *BTRCP*, and *ZNF217*. Significant associations with pCR were observed for *AXL*, *FGFR1*, *YAP*, and *BRCA1*. Low *AXL* and *BRCA1* expression levels were independently associated with pCR. In addition, their combined low expression was associated most strongly with breast pCR in this cohort. These findings should be interpreted as exploratory and require validation in independent cohorts.

## 1. Introduction

Chemotherapy has significantly improved overall survival in breast cancer, whether administered in adjuvant or neoadjuvant settings [1]. In particular, findings from the NSABP B-18 trial demonstrated that neoadjuvant chemotherapy (NAC) increases the rate of breast-conserving surgery by downstaging tumors while maintaining survival benefits comparable to those of adjuvant treatment [2]. Beyond its clinical advantages, NAC offers a unique opportunity to evaluate tumor biology and chemosensitivity in vivo.

Pathological complete response (pCR) has become a key endpoint in neoadjuvant clinical research, with several studies demonstrating its strong association with improved outcomes following NAC [3,4,5]. Consequently, there is growing interest in identifying factors linked to pCR, including classical pathological features such as tumor histology, grade, lymph node involvement, hormone receptor status, and HER2 expression, all of which have been correlated with NAC response [5]. Furthermore, gene expression profiling has highlighted the heterogeneous nature of breast cancer, revealing diverse molecular tumor profiles with differing chemosensitivities and outcomes [6]. Rouzier et al. reported that breast cancer molecular subtypes respond differently to NAC, with basal-like and HER2-positive tumors achieving 45% pCR, compared to only 6% in luminal tumors [7].

Tumor progression involves complex biological mechanisms and interconnected signaling pathways that influence therapeutic response [8]. Additionally, genes that mediate treatment sensitivity or resistance are differentially expressed across tumor subtypes [9,10]. For instance, the mTOR/PI3K pathway is linked to taxane response, while genes involved in DNA repair, such as *BCL2* and *ERCC4*, correlate with resistance to anthracyclines [10].

Given the large number of genes potentially involved in breast cancer biology, we selected a focused panel of candidate genes based on prior evidence of their involvement in tumor progression, molecular subtype differentiation, and mechanisms potentially related to response to chemotherapy. In this study, we analyzed the expression of 13 genes (*BRCA1*, *RAP80*, *FGFR1*, *GAS6*, *AXL*, *YAP*, *BTRCP*, *PTPN12*, *EZH2*, *HIF-1alpha*, *BIM*, *ROR1*, *ZNF217*) in a well-characterized cohort of patients treated with NAC. Our objective was to identify potential associations between gene expression, breast cancer subtypes, pCR, risk of recurrence, and clinical outcomes.

## 2. Results

### 2.1. Patients’ Characteristics

We included 92 patients. Their characteristics and tumor features are summarized in Table 1. The mean age at diagnosis was 48 years (range 31–68), with 67 patients (72.8%) being premenopausal. The median tumor size was 3.1 cm (range 0–8) on ultrasound and 3.6 cm (range 1–8) on magnetic resonance imaging. Lymph node involvement was identified in 53 patients (57.6%).

The majority of tumors were invasive ductal carcinoma (82 cases, 90.1%), grade II or III (56 cases, 61.6%), positive for hormone receptors (74 patients, 80.4%), and HER2-negative (64 patients, 69.6%). Tumor subtypes were distributed as follows: HR-positive/HER2-negative in 53 patients (57.6%), HER2-positive in 28 patients (30.4%), and triple-negative in 11 patients (12%). Based on ER, PR, HER2 status, and Ki67%, the patients were further classified into luminal A (23 patients, 25%), luminal B/HER2-negative (30 patients, 32%), luminal B/HER2-positive (21 patients, 23%), HER2-positive/HR-negative (7 patients, 8%), and triple-negative (11 patients, 12%).

A good pathological response (Miller–Payne [MP] classification grade 4/5) was achieved in 47 patients (51%), with breast pCR (MP grade 5) observed in 26 patients (28%).

### 2.2. Association of Gene Expression and Tumor Subtypes

**Figure 1:**
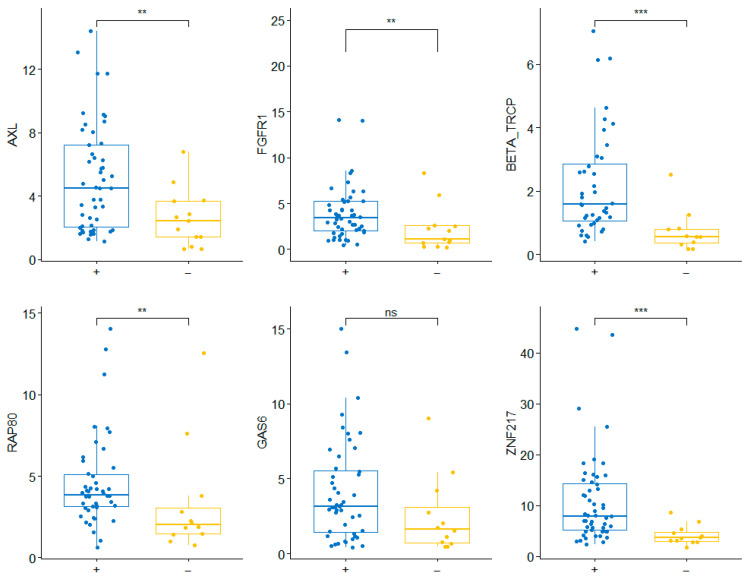
Association of molecular results with hormone receptor status (ns: *p* > 0.05; **: <0.01; ***: < 0.001).

**Figure 2:**
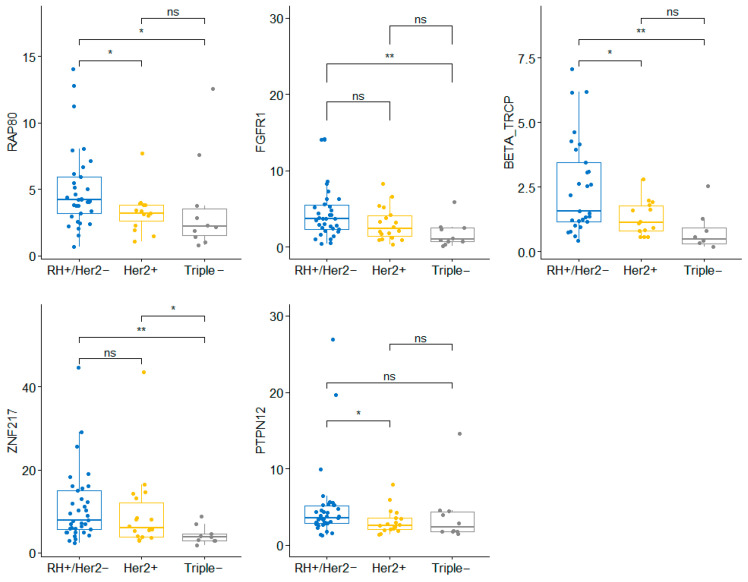
Association of molecular results with tumor subtype (ns: *p* > 0.05; *: <0.05; **: <0.01).

Medians and percentiles of mRNA expression were calculated for each gene in the 92 samples assessed (Supplementary Materials Table S1). The mRNA levels of certain genes were statistically significantly associated with tumor subtypes (Table 2, Figure 1). Positive-HR tumors showed high levels of *AXL* (M2 in 58% vs. 15%, *p* = 0.006), *FGFR1* (M2 in 58% vs. 15%, *p* = 0.006), *RAP80* (T2 + T3 in 54% vs 25%, *p* = 0.007), *GAS6* (M2 56% vs. 25%, *p* = 0.05), *BTRCP* (M2 in 60% vs. 9%, *p* = 0.003), and *ZNF217* (T3 in 42% vs. 0%, *p* = 0.005). Box plots are presented in Figure 1. When tumors were compared across the three main clinically used categories (HR-positive/HER2-negative, HER2-positive, and triple-negative), we found statistically significant differences in *RAP80* (T3 in 24% HR + /HER2− tumors, 27% in HER2+ and 70% in triple-negative, *p* = 0.022), *FGFR1* (M2 in 62%, 44% and 10%, respectively, *p* = 0.012), *BTRCP* (T3 in 48%, 14% and 12%, *p* = 0.013), *ZNF217* (T3 in 46%, 28% and 0%, *p* = 0.016) and *PTPN12* (T2 + T3 in 82%, 44% and 50%, *p* = 0.011) (Figure 2). Finally, the comparison of luminal A versus non-luminal A tumors revealed significantly high levels of *AXL* (*p* = 0.017), *FGFR1* (*p* = 0.022), *YAP* (*p* = 0.003), *GAS6* (*p* = 0.05), *BTRCP* (*p* = 0.003), and *PTPN12* (*p* = 0.029). In contrast, low levels of *ZNF217* (*p* = 0.016) and *RAP80* (*p* = 0.004) were found in HER2+/HR− and triple-negative tumors (Table 3). No statistically significant differences were observed in any case for *BRCA1* (*p* = 0.16), *BIM* (*p* = 0.38), *EZH2* (*p* = 0.3), *ROR1* (*p* = 0.47), and *HIF-1alpha* (*p* = 0.13).

### 2.3. Gene Expression Associated with pCR

**Figure 3:**
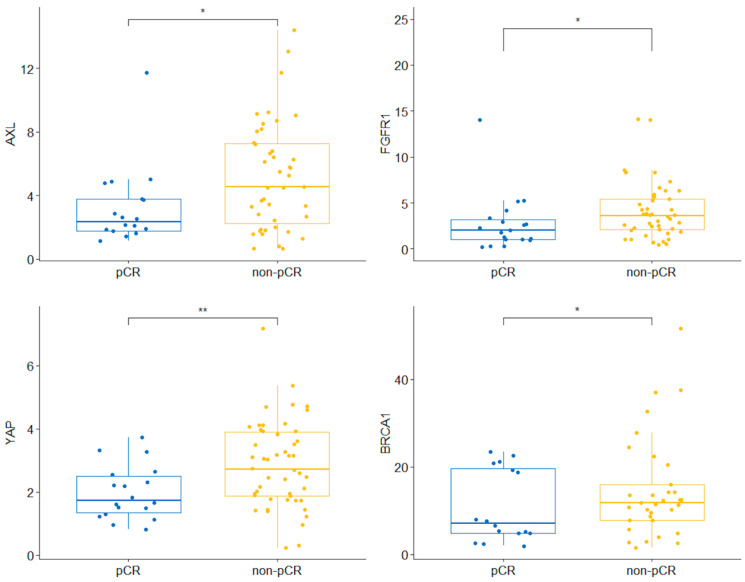
Association of gene expression with pathological response (*: <0.05; **: <0.01).

We observed a statistically significant association with pCR of the mRNA expression levels of *AXL* (T1 + T2 with pCR, *p* = 0.003), *FGFR1* (T1 was associated with pCR, *p* = 0.04), *YAP* (T1 was associated with pCR, *p* = 0.023), and *BRCA1* (T1 was associated with pCR, *p* = 0.027) (Figure 3, Table 3). A multivariable analysis of pCR was performed, adjusted for gene expression, lymph node involvement, and tumor subtype. We observed that only *AXL* and *BRCA1* mRNA levels were independent factors associated with pathological response, with odds ratios (ORs) of 14.03 (95% CI 1.44–136.61) for T1 + T2 of *AXL* and 7.07 (95% CI 1.41–35.43) for T1 of *BRCA1* (Table 4). However, confidence intervals were wide, likely reflecting limited sample size and event numbers; therefore, the magnitude of these estimates should be interpreted with caution. An analysis combining gene expression levels was also performed. Several 2- and 3-gene combinations were associated with breast pCR in this cohort; however, these analyses were based on very small subgroups and should be considered exploratory. For example, low *YAP* and *FGFR1* expression was associated with breast pCR in 62.5% of cases (n = 5), *p* = 0.038; low *BRCA1* and *AXL* expression was observed in all breast pCR cases within that subgroup (n = 6), *p* < 0.001; low *BRCA1* and *FGFR1* in 71.4% (n = 5), *p* = 0.018; low BRCA1 and YAP in 70% (n = 7), *p* = 0.005; and low *BRCA1*, *AXL* and *YAP* in all breast pCR cases within that subgroup (n = 5), *p* = 0.001.

### 2.4. Association of Gene Expression with PFS and OS

In the entire cohort, median follow-up was 40.07 months (IQR, 20.80–59.63 months); progression-free survival (PFS) at 60 months was 80.8% (95% CI = 70.4–91.2%), and overall survival (OS) at 60 months was 80.1% (95% CI = 65.7–94.4%) (Supplementary Materials Figures S1 and S2). Low *FGFR1* and *HIF-1alpha* mRNA levels were significantly associated with a longer OS. Median OS for high (M2) *FGFR1* levels was 101 months, compared to not reached for low (M1) levels (*p* = 0.043). *HIF-1alpha* at T1 showed a trend toward better OS, with a median of 101 months in T2 + T3, whereas it was not reached in T1 (*p* = 0.071). High levels of *BTRCP* were associated with a lower risk of relapse and longer overall survival (*p* = 0.049 and *p* = 0.06, respectively). *BTRCP* levels at T2 + T3 showed a tendency toward longer overall survival, with a median OS of 63 months in the T1 group versus not reached in the T2 + T3 group (*p* = 0.06). Regarding PFS, T2 + T3 levels of *BTRCP* were associated with a lower risk of relapse, with a 56% rate in T1 versus 89% in T2 + T3 (*p* = 0.049; Supplementary Materials Figures S3–S6).

## 3. Discussion

Breast tumors present considerable heterogeneity, leading to significant differences in chemosensitivity. The identification and application of specific predictive factors in pretreatment biopsies can help ensure that only patients who truly benefit receive neo/adjuvant treatment. Clinical and classical pathological factors are currently used to predict pCR in routine practice [11]. However, it is well known that the cell cycle is controlled by the balance among oncogenes, tumor suppressor genes, and genes involved in DNA repair. Genetic alterations in some of them can dysregulate cell signaling pathways, promote carcinogenesis, and alter the effect of chemotherapeutic drugs. Therefore, a molecular approach could identify more specific predictive markers of response than pathological factors.

With this aim, we analyzed the mRNA expression levels of 13 candidate genes in a series of 92 patients treated with NAC. According to several studies, each breast tumor subtype can express different molecular profiles [7,12]. Our series also revealed distinct gene expression patterns across tumor subtypes. Similar to findings reported by Littlepage et al., we found high *ZNF217* mRNA levels in positive-HR tumors and low expression in triple-negative tumors [13]. The genes *AXL*, *FGFR1*, *RAP80*, *GAS6*, and *BTRCP* were overexpressed in positive-HR tumors. In contrast, triple-negative and HER2+/HR− tumors were associated with low *RAP80* mRNA levels. In addition, we clearly identified higher levels of *FGFR1*, *PTPN12*, and *BTRCP* in luminal tumors compared with non-luminal tumors. These data are consistent with previous studies in other tumor types [12,13,14,15,16]. For example, *AXL* and *GAS6* overexpression has been found in hormone-sensitive endometrial and prostate carcinomas [17,18]. It becomes important as these genes also have a thrombotic effect, and this subgroup of patients already presents an increased risk of thrombosis due to tamoxifen and chemotherapy. So, a targeted treatment blocking *AXL* and *GAS6* could benefit these patients by reducing the risk of thrombosis [19]. The genes mentioned above (*AXL*, *FGFR1*, *YAP*, *BTRCP*, *PTPN12*, *RAP80*) could help refine molecular subtypes in clinical practice.

*YAP* overexpression was observed in 93% of luminal A tumors, compared with 38% in luminal B/HER2-negative tumors (*p* = 0.003). *YAP* is considered an ER and PR coactivator, which explains the significant association between reduced *YAP* expression and negative-HR tumors [20]. Moreover, our results confirmed that *YAP* levels were also inversely correlated with HER2 overexpression. Several studies have reported the importance of *BRCA1* in DNA repair and its implication in sporadic breast cancer due to “dysfunction”. Low expression of *BRCA1* and *RAP80* has been associated with high-grade carcinomas that lack hormone receptor or HER2 expression [21,22]. In our series, an association with RAP80 was observed, but no differences in *BRCA1* levels were observed.

As mentioned, the main aim of our study was to correlate the mRNA levels of several genes with different chemotherapy response patterns, particularly breast pCR. Ignatiadis et al. showed that distinct molecular pathways were associated with pCR across molecular subtypes in the largest series published to date (996 patients and 17 genes studied) [9]. In our analysis, we identified an association between low *BRCA1* expression and pCR. Although the prognostic impact of *BRCA1* in breast cancer has been largely recognized in clinical studies, its predictive value has been little studied. *BRCA1* is a well-known tumor suppressor gene involved in DNA repair. Consequently, *BRCA1* over- or under-expression may significantly affect the response to NAC in preclinical models (Margely et al.) and in patients treated with DNA-damaging agents, such as anthracyclines or cisplatin [23,24]. However, controversial data still exist, such as the study by Nakai et al., which failed to correlate *BRCA1* levels with pCR in a series of 32 tumor samples [25]. In our study, low *FGFR1* expression levels (T1) were correlated with pCR (*p* = 0.04), a result that contradicted the study by Massabeau et al., which suggested that *FGFR1*-negative tumors were associated with lower pCR [26]. Our findings can be explained by the overexpression of *FGFR1* by luminal tumors, which are associated with poor chemosensitivity. In lung cancer, it has been suggested that tumors with amplification of *FGFR1* present a poor DFS and high recurrence risk [27]. In breast cancer, such overexpression might promote resistance to endocrine therapy through a progesterone receptor suppression [15].

Regarding survival, our study showed significantly improved OS in patients with low *FGFR1* levels (*p* = 0.043) and a trend toward improved OS with low T1 *HIF-1alpha* and T2 + T3 *BTRCP* (*p* = 0.071 and 0.06, respectively). *BTRCP* levels at T2 + T3 were also associated with a significantly increased PFS (*p* = 0.049). There is limited information about the *BTRCP* gene, and its role as a prognostic factor remains unclear. Some studies have reported that high levels of *BTRCP* might block *VEGFR2* and inhibit angiogenesis, suggesting it could be a marker of good prognosis [28]. In our series, overexpression was associated with better OS and DFS. Regarding *HIF-1alpha*, hypoxic adaptation is vital for tumor progression, and the hypoxic response is mediated by *HIF-1alpha*. It stimulates angiogenesis, which sustains tumor growth, so overexpression is associated with an adverse outcome [29]. In our study, high levels were associated with poorer OS.

Our study has some limitations. First, pCR was defined according to the MP grading system (MP5), reflecting the absence of residual invasive tumor cells in the breast specimen (breast pCR). Nodal status after NAC was not incorporated into this definition. This approach differs from the contemporary ypT0/is ypN0 definition commonly used in neoadjuvant trials, and therefore, comparisons with studies that apply standard ypT0/is ypN0 criteria should be interpreted with caution. In addition, although 71% of patients received an anthracycline/taxane-based chemotherapy, our cohort was not completely homogeneous regarding neoadjuvant treatment. The study period (2000–2011) spans years during which treatment strategies evolved, including the introduction of trastuzumab in the neoadjuvant setting after 2006. Consequently, not all HER2-positive patients received trastuzumab, which may have influenced pCR rates in this subgroup and should be considered when interpreting the results. In addition, multiple comparisons were performed across 13 genes and several subgroup and combination analyses, increasing the risk of false-positive findings. Finally, multivariable models may be prone to overfitting given the limited number of events, as suggested by wide confidence intervals; therefore, these findings should be considered hypothesis-generating and require validation in larger, independent cohorts.

Despite these limitations, our data suggest that the study of molecular markers may be useful for defining specific tumor subtypes and predicting prognosis. In this context, some of the molecular alterations studied in our article provide evidence that the analysis and application of these markers can play an important role in more effective, personalized therapies. Luminal tumors were characterized by high levels of *FGFR1*, *BTRCP*, *ZNF217*, and *PTPN12*. Low *RAP80* levels were more frequently observed in HER2-positive and triple-negative tumors. Consistently, high levels of *AXL*, *FGFR1*, *YAP*, and *PTPN12* were associated with poor pathological response, whereas low *AXL* and *BRCA1* expression levels were associated with pCR. In the multivariable analysis, *AXL* and BRCA1 were the only genes independently associated with pCR (AXL: OR 14.03; BRCA1: OR 7.07), although the wide confidence intervals suggest that these findings should be interpreted with caution. These results were confirmed in the signature analysis, with the combination of *AXL* and *BRCA1* mRNA levels showing the strongest association with pCR (*p* < 0.001). Taken together, these exploratory results suggest that combined *AXL* and *BRCA1* expression may be associated with breast pCR beyond immunohistochemistry-defined subtype and merit validation in larger, uniformly treated, independent cohorts.

## 4. Materials and Methods

### 4.1. Participants

We retrospectively reviewed NAC experience at our center from January 2000 to January 2011, analyzing gene expression profiles from 92 tumor pre-treatment core biopsies in the prospective Breast Cancer Database (Supplementary Materials Table S2). We included patients with T1c-T4/N0-N1 tumors who were deemed candidates for NAC, as determined by our institution’s Breast Cancer Multidisciplinary Meeting. Patients with metastatic disease, bilateral breast cancer, or male breast cancer were excluded. Clinical and pathological data were collected, including patient age, tumor size, lymph node involvement, histological type, tumor grade, hormone receptor (HR), and HER2 status. Pretreatment tumor size was evaluated through clinical examination and radiographic measurements. Associations were analyzed between gene expression levels, specific tumor subtypes, pathological response, and long-term outcomes.

This study adhered to the principles of the Declaration of Helsinki and was conducted under an approved institutional review board protocol at Quirón Hospitals. Written informed consent was obtained from all participants and adequately documented; all samples were de-identified to ensure confidentiality. The manuscript was prepared in accordance with the REMARK guidelines for tumor marker prognostic studies [30].

### 4.2. Treatment and Pathological Assessment

An anthracycline-based scheme, with or without a taxane, was used. Since 2006, trastuzumab has been included in the neoadjuvant treatment of HER2-positive patients; treatment was halted at the time of surgery and resumed as an adjuvant therapy for one year in HER2-positive tumors. All drugs were administered intravenously. Patients underwent either mastectomy or breast-conserving surgery, depending on their tumor response to NAC. Following surgery, adjuvant treatments—including breast radiotherapy, regional nodal radiation, hormone therapy, or adjuvant chemotherapy—were administered according to institutional guidelines. Pathological local response was assessed in surgical specimens using the MP classification [31]. Postoperative tumor size was not used as an endpoint in this analysis. All tumor specimens were re-evaluated by an expert pathologist for quantification using the MP scale. Pathological response was categorized as good (MP 4 and 5) or poor (MP 1–3). For the purposes of this study, pCR was defined as MP grade 5, indicating the absence of residual invasive tumor cells in the breast specimen (breast pCR). Nodal status after NAC was not incorporated into this definition.

### 4.3. Immunohistochemical Staining and Definition of Tumor Subtypes

Hormone receptor status, HER2 status, and Ki67 value were analyzed on pretreatment core biopsies. Determination of estrogen receptors (ER) (clone 6F11, Novocastra 1/200, UK), progesterone receptors (PR) (clone 1A6, Novocastra 1/200, UK), and Her2 (Clone CB11, Oracle, Leica Biosystems^®^, Deer Park, IL, USA) was performed by immunohistochemistry using, in all cases, the manufacturer’s pre-diluted antibody. According to standardized guidelines, ER and PR were considered positive when ≥10% of tumor cell nuclei were stained. Positive HER2 was considered as strong overexpression (3+) or equivocal overexpression (2+) if the FISH technique (HER-2 DNA PathVysion, Abbott^®^, Abbott Park, IL, USA) was positive. All other staining patterns were interpreted as negative (0/1+). Ki67 was assessed by immunohistochemistry using MIB-1 antibodies; Ki67 values were defined as low (<14%) and high (≥14%). Molecular tumor subtypes were categorized according to the new immunohistochemistry correlation published by Prat et al. [32]: luminal A (ER-positive/PR > 20%/HER2-negative/Ki67 < 14%), luminal B/HER2− (ER+/PR < 20%/HER2-/Ki67 > 14%), luminal B/HER2+ (ER+/PR+/HER2+), positive-HER2 (ER−/PR−/HER2+), and triple-negative (ER−/PR−/HER2−).

### 4.4. Gene Expression Analysis Using Quantitative Reverse Transcription Polymerase Chain Reaction

Formalin-fixed paraffin-embedded tissue (FFPET) slides (4 µm) were obtained by standard procedures and stained with hematoxylin and eosin. A pathologist determined the tumor areas and evaluated the percentage of tumor infiltration. According to the manufacturer’s instructions, RNA was extracted from the selected areas using a high-purity FFPET RNA isolation kit (Roche Diagnostics, Mannheim, Germany). Complementary DNA (cDNA) was synthesized using the M-MLV Reverse Transcriptase Enzyme (Thermo Fisher Scientific, Waltham, MA, USA). Hereafter, cDNA was added to Taqman Universal Master Mix (Applied Biosystems, Thermo Fisher Scientific, Waltham, MA, USA) in a 12.5 μL reaction with specific primers and probes for each gene. The primer and probe sets were designed using Primer Express 3.0 Software (Applied Biosystems, Thermo Fisher Scientific, Waltham, MA, USA) based on their RefSeq entries (http://www.ncbi.nlm.nih.gov/LocusLink, accessed on 10 January 2014). The mRNA levels of the following genes were analyzed: *BRCA1*, *RAP80*, *FGFR1*, *GAS6*, *AXL*, *YAP*, *BTRCP*, *PTPN12*, *EZH2*, *HIF-1alpha*, *BIM*, *ROR1*, and *ZNF217*. These genes were selected a priori based on previously reported associations with breast cancer biology, molecular subtype differentiation, and mechanisms potentially involved in response or resistance to chemotherapy. Gene-specific primers are presented in Supplementary Materials Table S3. Quantification of gene expression was performed using the ABI Prism 7900HT Sequence Detection System (Applied Biosystems, Thermo Fisher Scientific, Waltham, MA, USA). Levels of mRNA were expressed as arbitrary units based on the CT values. Gene expression levels were normalized to internal reference controls and analyzed using Ct values obtained from qRT-PCR assays performed in replicate. Samples with insufficient RNA quality or low tumor cellularity were excluded from the analysis. Commercial RNAs were used as controls (liver and lung; Stratagene, Agilent Technologies, La Jolla, CA, USA). In all quantitative experiments, a sample was considered not evaluable when the standard deviation of the Ct values was >0.30 in 2 independent analyses.

### 4.5. Statistical Analysis

Quantitative variables were compared with the Wilcoxon Mann–Whitney or Student’s *t*-test, and categorical variables with the Pearson chi-square or Fisher’s exact test. Gene expression values were categorized using either median or tertile distributions, depending on the variability in gene expression. When subgroup sizes were small, adjacent tertile categories were combined to improve statistical stability. PFS was calculated from the date of diagnosis until the earliest of progression or death from any cause. OS was calculated from the diagnosis to death from any cause. Survival was estimated using Kaplan–Meier curves, and differences were assessed using the log-rank test.

The following variables were used to predict prognostic factors for NAC: hormone receptor status, T stage, lymph node involvement, age, histologic grade, and tumor subtype. A univariate analysis was performed for each variable using the log-rank test. A multivariable analysis was conducted using a logistic regression model to assess the adjusted influence of specific gene expression and the pathological response. All statistical analyses were performed using R software version 3.2.2 (R Foundation for Statistical Computing, Vienna, Austria). All tests were two-sided, and the significance level was set at 0.05.

## 5. Conclusions

In this study, distinct gene expression patterns were observed across breast cancer molecular subtypes. High expression of *AXL*, *FGFR1*, *RAP80*, *GAS6*, *BTRCP*, and *ZNF217* was associated with hormone receptor-positive tumors. Regarding treatment response, low *AXL* and *BRCA1* expression levels were independently associated with pCR in the multivariable analysis. These findings suggest that gene expression profiling may help to identify biomarkers associated with response to NAC. However, given the limited sample size and exploratory nature of the analyses, these results require validation in larger independent cohorts.

## Figures and Tables

**Table 1:** Patient and tumor characteristics.

	Patients with RNA Assessment (%) (Range) (*N* = 92)
Age (mean years)	47.57 (31–68)
Hormonal status premenopausal menopausal	67 (72.8) 25 (27.2)
Tumor size (median cm) Exploration Ultrasound Magnetic resonance imaging	3.48 (0–10) 3.11 (0–8) 3.63 (1–8)
Lymph node involvement	53 (57.6)
Tumor histology Invasive ductal carcinoma Invasive lobular carcinoma Others NA	82 (90.1) 7 (7.7) 2 (2.2) 1
Pre-chemotherapy tumor grade I II III NA	35 (38.5) 37 (40.7) 19 (20.9) 1
HR+	74 (80.4)
ER+	70 (76.1)
PR+	60 (65.2)
HER2-Positive	28 (30.4)
Ki 67 High (> o = 14%) Low (<14%) NA	54 (66.7) 27 (33.3) 11
pCR MP 5	26 (28.3)
Tumor subtype Positive-HR/negative-HER2 HER2-positive Triple-negative	53 (57.6) 28 (30.4) 11 (12)
Molecular subtype Luminal A Luminal B HER2− Luminal B HER2+ HER2+/RH− Triple-Negative	23 (25) 30 (32.6) 21 (22.8) 7 (7.6) 11 (12)

NA: not assessed; HR: hormonal receptors; ER: estrogen receptors; PR: progesterone receptors.

**Table 2:** Gene expression levels according to tumor subtypes defined by immunohistochemistry.

RNA Expression Levels %—Cases													
	BRCA1 T1	RAP80 T1	BIM T1	EZH2 T1	ROR1 T1	FGFR1 M2	PTPN12 T1	YAP M2	GAS6 M2	BTRCP T1	HIF-1α T3	ZNF217 T1	AXL M2
HR+	29 12/41	22 10/46	31 15/49	32 12/37	32 8/25	58 30/52	28 15/53	55 30/55	56 27/48	20 8/40	31 15/48	23 12/52	58 28/48
HR−	62 5/8	75 9/12	50 6/12	40 4/10	33 3/9	15 2/13	54 7/13	31 4/13	25 3/12	82 9/11	33 4/12	75 9/12	15 2/13
*p*	0.16	<0.001	0.32	0.66	0.99	0.006	0.16	0.12	0.05	0.003	0.89	0.005	0.006
HR+ HER2−	32 9/28	24 8/33	26 9/35	24 6/25	26 5/19	62 23/37	18 7/38	59 24/41	63 22/35	17 5/29	24 8/33	22 8/37	57 20/35
HER2+	31 5/16	27 4/15	41 7/17	43 6/14	50 4/8	44 8/18	56 10/18	35 6/17	31 5/16	43 6/14	50 9/18	33 6/18	50 8/16
TN	60 3/5	70 7/10	56 5/9	50 4/8	29 2/7	10 1/10	50 5/10	40 4/10	33 3/9	75 6/8	22 2/9	79 7/9	20 2/10
*p*-value	0.72	0.001	0.38	0.3	0.79	0.012	0.011	0.21	0.06	0.013	0.36	0.016	0.11
Total	49	58	61	47	34	65	66	68	60	51	60	64	61

T: tercile; M: median; HR: hormonal receptors; TN: triple-negative.

**Table 3:** Gene expression levels across molecular subtypes and their association with pCR.

RNA Expression Levels %—Cases													
	BRCA1 T1	RAP80 T1	BIM T1	EZH2 M2	ROR1 T1	FGFR1 T1	PTPN12 T1	YAP M2	GAS6 M2	BTRCP T1	HIF-1α T3	ZNF217 T1	AXL T1 + T2
LA	25 2/8	8 1/12	23 3/13	56 5/9	17 1/6	21 3/14	7 1/15	93 14/15	79 11/14	73 8/11	36 4/11	21 3/14	31 4/13
LB HER2−	35 7/20	33 7/21	27 6/22	62 10/16	31 4/13	17 4/23	26 6/23	38 10/26	52 11/21	33 6/18	18 4/22	22 5/23	73 16/22
LB HER2+	23 3/13	15 2/13	43 6/14	42 5/12	50 3/6	47 7/15	53 8/15	43 6/14	38 5/13	18 2/11	47 7/15	27 4/15	69 9/13
HER2+ HR−	67 2/3	100 2/2	33 1/3	50 1/2	50 1/2	67 2/3	67 2/3	00/3	00/3	00/3	67 2/3	67 2/3	100 3/3
TN	60 3/5	70 7/10	56 5/9	37 3/8	29 2/7	60 6/10	50 5/10	40 4/10	33 3/9	12 1/8	22 2/9	78 7/9	90 9/10
*p*	0.5	<0.001	0.79	0.7	0.9	0.05	0.02	0.003	0.05	0.003	0.13	0.016	0.017
Total	49	58	61	47	34	65	66	68	60	51	60	64	61
*pCR* *(MP 5)*	*53* *9/17*	*32* *6/19*	*43* *9/21*	*40* *6/24*	*27* *3/11*	*45* *10/22*	*41* *9/22*	*15* *5/34*	*20* *6/30*	*18* *3/17*	*37* *7/19*	*28* *6/21*	*95* *19/20*
*p*-value	0.027	0.07	0.059	0.69	0.72	0.04	0.12	0.028	0.15	0.37	0.55	0.74	0.003

T: tercile; M: median; LA: luminal A; LB: luminal B; HR: hormonal receptors; TN: triple-negative.

**Table 4:** Gene expression levels and predictive factors for pCR.

	Odds Ratio	95% CI	*p*-Value
AXL expression	14.03	1.44–136.61	0.023
BRCA1 expression	7.07	1.41–35.43	0.017
FGFR1 expression	2.2	0.61–7.92	0.22
YAP expression	3	0.83–10.75	0.09

## Data Availability

The original contributions presented in this study are included in the article/Supplementary Material. Further inquiries can be directed to the corresponding author.

## Supplementary Materials

The following supporting information can be downloaded at https://www.mdpi.com/article/10.3390/ijms27062564/s1.

## Abbreviations

The following abbreviations are used in this manuscript:

NAC	Neoadjuvant chemotherapy
pCR	Pathological complete response
MP	Miller–Payne
ER	Estrogen receptors
PR	Progesterone receptors
FFPET	Formalin-fixed paraffin-embedded tissue
cDNA	Complementary DNA
PFS	Progression-free survival
OS	Overall survival
ORs	Odds ratios
