# Supplementary material for: Gene Expression Profiles Associated with Molecular Subtypes and Pathological Response to Neoadjuvant Treatment in Surgical Breast Cancer
Source: Int J Mol Sci. 2026 Mar 11;27(6):2564. doi: 10.3390/ijms27062564 (PMC13027109; doi:10.3390/ijms27062564)
Supplement: Supplementary file 1 [file ijms-27-02564-s001.zip › ijms-4186807-supplementary.pdf]

Supplementary Materials - Tables

Table S1. Pre-treatment samples

ID PANGAEA	Nº BIOPSY	ORGAN	EXTERNAL DIAGNOSIS	QUANTITY	QUALITY	RNA/DNA	AP Evaluation	% TI	% S	% N	% IC	Type of dissection
**MC1**	99-287	Breast	-	adec	val	-	Infiltrating Carcinoma	30	70	5	20	MICRO
**MC2**	00-978	Breast	-	adec	val	-	Infiltrating Carcinoma	30	70	0	5	MACRO
**MC3**	00-1102	Breast	-	adec	val	-	Infiltrating Carcinoma	60	40	0	10	MICRO
**MC4**	00-1582	Breast	-	adec	val	-	Infiltrating Carcinoma	70	30	0	10	MACRO
**MC5**	00-4732	Breast	-	scant	val	NO RNA	Infiltrating Carcinoma	15	85	0	10	MICRO
**MC6**	01-1921 B	Breast	-	scant	val	-	Infiltrating Carcinoma	20	80	0	10	MICRO
**MC7**	01-2649	Breast	-	adec	val	-	Infiltrating Carcinoma	70	30	0	20	MICRO
**MC8**	01-3592	Breast	-	scant	val	NO RNA	Infiltrating Carcinoma	30	70	0	20	MICRO
**MC9**	02-897	Breast	-	scant	val	NO RNA	Infiltrating Carcinoma	70	30	0	5	MACRO
**MC10**	02-1812	Breast	-	adec	val	-	Infiltrating Carcinoma	80	20	0	10	MACRO
**MC11**	03-2878	Breast	-	adec	val	-	Infiltrating Carcinoma	60	40	0	10	MICRO
**MC12**	03-4353	Breast	-	adec	val	-	Infiltrating Carcinoma	80	20	5	15	MICRO
**MC13**	04-161	Breast	-	adec	val	-	Infiltrating Carcinoma	70	30	0	10	MICRO
**MC14**	05-4059	Breast	-	adec	val	-	Infiltrating Carcinoma	80	20	0	10	MACRO
**MC15**	05-4803	Breast	-	scant	val	NO RNA	Infiltrating Carcinoma	35	65	0	15	MICRO
**MC16**	06-513	Breast	-	scant	val	-	Infiltrating Carcinoma	40	60	0	20	MICRO
**MC17**	06-3647	Breast	-	adec	val	-	Infiltrating Carcinoma	60	40	0	10	MICRO
**MC18**	06-7657	Breast	-	adec	val	-	Infiltrating Carcinoma	40	60	5	30	MICRO
**MC19**	07-114	Breast	-	adec	val	-	Infiltrating Carcinoma	50	50	0	15	MICRO
**MC20**	07-946	Breast	-	scant	val	-	Infiltrating Carcinoma	30	70	0	10	MICRO
**MC21**	07-1173	Breast	-	adec	val	-	Infiltrating Carcinoma	80	20	5	10	MACRO
**MC22**	07-4669	Breast	-	adec	val	-	Infiltrating Carcinoma	80	20	20	20	MICRO
**MC23**	07-5436	Breast	-	adec	val	-	Infiltrating Carcinoma	30	70	0	10	MICRO
**MC24**	07-8618	Breast	-	adec	val	-	Infiltrating Carcinoma	20	80	0	10	MICRO
**MC25**	07-8645	Breast	-	adec	val	-	Infiltrating Carcinoma	40	60	0	10	MICRO
**MC26**	08-950	Breast	-	adec	val	-	Infiltrating Carcinoma	40	60	0	10	MICRO

**MC27**	08-2063	Breast	-	adec	val	-	Infiltrating Carcinoma	20	80	0	10	MACRO
**MC28**	08-4730	Breast	-	scant	val	-	Infiltrating Carcinoma	70	30	5	15	MICRO
**MC29**	08-6841	Breast	-	adec	val	-	Infiltrating Carcinoma	60	40	5	10	MICRO
**MC30**	08-7497	Breast	-	adec	val	-	Infiltrating Carcinoma	60	40	0	10	MICRO
**MC31**	08-8244	Breast	-	scant	val	-	Infiltrating Carcinoma	60	40	0	10	MICRO
**MC32**	08-8568	Breast	-	adec	val	-	Infiltrating Carcinoma	20	80	0	5	MACRO
**MC33**	08-9069	Breast	-	adec	val	-	Infiltrating Carcinoma	35	65	5	10	MICRO
**MC34**	08-9369	Breast	-	adec	val	-	Infiltrating Carcinoma	90	10	0	10	MICRO
**MC35**	09-439	Breast	-	scant	val	-	Infiltrating Carcinoma	20	80	5	40	MICRO
**MC36**	09-3670	Breast	-	adec	val	-	Infiltrating Carcinoma	40	60	5	15	MACRO
**MC37**	09-3757	Breast	-	adec	val	-	Infiltrating Carcinoma	80	20	0	5	MACRO
**MC38**	09-5058	Breast	-	adec	val	-	Infiltrating Carcinoma	70	30	0	10	MACRO
**MC39**	09-5916	Breast	-	adec	val	-	Infiltrating Carcinoma	60	40	0	10	MICRO
**MC40**	09-6060	Breast	-	adec	val	-	Infiltrating Carcinoma	60	40	0	10	MICRO
**MC41**	09-6404	Breast	-	adec	val	-	Infiltrating Carcinoma	30	70	0	10	MICRO
**MC42**	09-6682	Breast	-	adec	val	-	Infiltrating Carcinoma	65	35	0	15	MICRO
**MC43**	09-7770	Breast	-	adec	val	-	Infiltrating Carcinoma	30	70	0	10	MACRO
**MC44**	10-4140	Breast	-	adec	val	-	Infiltrating Carcinoma	60	40	0	5	MACRO
**MC45**	10-4240	Breast	-	adec	val	-	Infiltrating Carcinoma	80	20	5	10	MACRO
**MC46**	10-4249	Breast	-	adec	val	-	Infiltrating Carcinoma	70	30	0	5	MACRO
**MC47**	10-4301	Breast	-	scant	val	-	Infiltrating Carcinoma	20	80	0	5	MICRO
**MC48**	10-7204	Breast	-	adec	val	-	Infiltrating Carcinoma	70	30	0	15	MACRO
**MC49**	10-8512	Breast	-	adec	val	-	Infiltrating Carcinoma	30	70	0	5	MACRO
**MC50**	11-978	Breast	-	adec	val	-	Infiltrating Carcinoma	80	20	0	5	MACRO
**MC51**	11-2317	Breast	-	adec	val	-	Infiltrating Carcinoma	90	10	0	5	MACRO
**MC52**	11-3455	Breast	-	adec	val	-	Infiltrating Carcinoma	70	30	0	15	MACRO
**MC53**	10-2214	Breast	-	adec	val	-	Infiltrating Carcinoma	35	65	0	10	MACRO
**MC54**	10-5101	Breast	-	adec	val	-	Infiltrating Carcinoma	70	30	10	10	MACRO
**MC55**	10-5692	Breast	-	adec	val	-	Infiltrating Carcinoma	80	20	0	5	MACRO
**MC56**	10-543 B	Breast	-	adec	val	-	Infiltrating Carcinoma	70	30	15	10	MACRO
**MC57**	09-9360	Breast	-	adec	val	-	Infiltrating Carcinoma	40	60	20	5	MICRO

**MC58**	10-912	Breast	-	adec	val	-	Infiltrating Carcinoma	80	20	0	5	MICRO
**MC59**	10-7180	Breast	-	adec	val	-	Infiltrating Carcinoma	85	15	0	5	MACRO
**MC60**	10-8639	Breast	-	adec	val	-	Infiltrating Carcinoma	70	30	0	20	MICRO
**MC61**	10-8712	Breast	-	adec	val	-	Infiltrating Carcinoma	70	30	0	0	MACRO
**MC62**	11-9577	Breast	-	adec	val	-	CDI	40	60	2	18	MICRO
**MC63**	11-8903	Breast	-	adec	val	-	CLI	40	60	-	-	MICRO
**MC64**	11-10016	Breast	-	adec	val	-	CDI	50	50	-	30	MICRO
**MC65**	11-1555	Breast	-	adec	val	-	CLI	30	70	-	-	MICRO
**MC66**	11-4692	Breast	-	adec	val	-	CDI	50	50	-	20	MACRO
**MC67**	00-693	Breast	-	adec	val	-	Infiltrating Carcinoma	30	70	0	30	MICRO
**MC68**	03-3750	Breast	-	scant	val	NO RNA	Infiltrating Carcinoma	20	80	0	15	MICRO
**MC69**	04-5158	Breast	-	scant	val	NO RNA/DNA	Infiltrating Carcinoma	10	90	0	5	MICRO
**MC70**	06-4932	Breast	-	scant	val	NO RNA	Infiltrating Carcinoma	10	90	0	10	MICRO
**MC71**	09-1251	Breast/Skin	-	scant	val	NO RNA	Infiltrating Carcinoma	10	90	0	10	MICRO
**MC72**	98-3712	Breast	-	scant	val	NO RNA	Infiltrating Carcinoma	40	50	0	10	MICRO
**MC73**	08-3709	Breast	-	adec	val	-	Infiltrating Carcinoma	70	30	0	10	MACRO
**MC74**	10-3093 B	Breast	-	adec	val	-	Infiltrating Carcinoma	70	30	0	10	MACRO
**MC75**	05-4344	Breast	-	-	-	-	Not Tumor	-	-	-	-	-
**MC76**	11-4396	Breast	-	adec	val	-	Infiltrating Carcinoma	60	40	10	10	MACRO
**MC77**	10-1853	Breast	-	adec	val	-	Infiltrating Carcinoma	40	60	0	5	MACRO
**MC78**	10-2334	Breast	-	adec	val	-	Infiltrating Carcinoma	50	50	0	10	MACRO
**MC79**	11-6523	Breast	-	adec	val	-	Infiltrating Carcinoma	40	60	0	5	MACRO
**MC80**	11-4135	Breast	-	adec	val	-	Infiltrating Carcinoma	90	10	20	10	MACRO
**MC81**	09-8129	Breast	-	scant	val	NO RNA	Infiltrating Carcinoma	40	60	0	5	MICRO
**MC82**	09-6586	Breast	-	adec	val	-	Infiltrating Carcinoma	70	30	10	10	MACRO
**MC83**	09-10526 A	Breast	-	scant	val	-	CDI	20	80	0	30	MICRO
**MC84**	12-2610	Breast	-	adec	val	-	Infiltrating Carcinoma	70	30	0	10	MICRO
**MC85**	12-8948	Breast	-	adec	val	-	Infiltrating Carcinoma	85	17	0	3	MICRO
**MC86**	12-5891 A1	Breast	-	adec	val	-	Infiltrating Carcinoma	90	10	-	-	MACRO
**MC87**	12-	Breast	-	adec	val	-	Infiltrating	90	10	-	-	MACRO

	5144						Carcinoma					
**MC88**	12-4194	Breast	-	adec	val	-	Infiltrating Carcinoma	87	10	-	3	MACRO
**MC89**	12-4820	Breast	-	adec	val	-	Infiltrating Carcinoma	87	10	-	3	MACRO
**MC90**	11-10818	Breast	-	adec	val	-	Infiltrating Carcinoma	90	10	-	-	MACRO
**MC91**	B11-3893	Breast	-	adec	val	-	Infiltrating Carcinoma	40	60	-	-	MACRO
**MC92**	12-5408	Breast	-	adec	val	-	CDI	45	55	0	0	MICRO

Table S2. List of primers and probes used in quantitative PCR

GENES	PRIMERS		PROBES
BRCA1	F	5′GGCTATCCTCTCAGAGTGACATTTTA 3′	6FAM 5′CCACTCAGCAGAGGG 3′ MGB
	R	5′GCTTTATCAGGTTATGTTGCATGGT 3′	
RAP80	F	5′ ACATCAAGTCTTCAGAAACAGGAGC 3′	6FAM 5′ TCAGGGTGCCTTCACCA 3′MGB
	R	5′ TGCAGCCTGCCTCTTCCAT 3′	
FGFR1	F	5′ CCACACTGCGCTGGTTGA 3′	6FAM 5′ AACCTGACCACAGAATT ′ MGB
	R	5′ GGCATAACGGACCTTGTAGCC 3′	
AXL	F	5′ CAGCGCAGCCTGCATGT 3′	6FAM 5′ CAGGGCTGAACAAGAC 3′ MGB
	R	5′ GCGTTATGGGCTTCGCAG 3′	
B-TRCP	**Hs01046327_m1**		
GAS 6	F	5′ TGGAGGAGCTGTGCAGCC 3′	6FAM 5′ TGTTCGAGAACGACCC 3′ MGB
	R	5′ CTTGGGTAAAAATAATCCGTCTCG 3′	
PTPN12	F	5′ ATATCCCACAGCCACTGGAG 3′	6FAM 5′ AAGAACAGATACAAGGACATAC ′ MGB
	R	5′ TCGGCTGTGATCAAATGGC 3′	
YAP	F	5′ TTGGGAGATGGCAAAGACATC 3′	6FAM 5′ TCAGAGATACTTCTTAAATCACA′ MGB
	R	5′ GCCATGTTGTTGTCTGATCGA 3′	
HIF1a	**Hs00153153_m1**		
EZH2	**Hs01016789_m1**		
ROR1	F	5′ CAAGTCCAGGATACTCAGATGAGTATG 3′	6FAM 5′ AGAAGATGGATTCTGTCAGC 3′ MGB
	R	5′ TGCACATGCAATCCCTCTGTA 3′	
ZNF217	**Hs00919915_m1**		
BIM	**Hs00708019_s1**		

F, Forward; R, Reverse

Table S3. Descriptive data for each gene

		AXL	*BRCA1*	*RAP80*	*BIM*	*EZH2*	*ROR1*	*FGFR1*	*PTPN12*	*YAP*	*GAS6*	*BTRCP*	*HIF1a*	*ZNF217*
Percentile	25	1.847	5.233	2.358	3.307	3.246	.974	1.576	2.364	1.674	1.181	.756	.609	4.064
	30	2.057	6.624	2.744	3.825	3.904	1.126	1.958	2.551	1.772	1.488	.811	.659	4.865
	33	2.283	7.610	2.976	4.107	3.992	1.256	2.116	2.714	1.866	1.574	.926	.698	4.914
	50	3.733	11.29	3.771	5.388	6.143	2.071	2.946	3.198	2.430	3.013	1.254	.901	6.856
	66	5.486	14.19	4.221	7.004	8.258	3.019	4.225	4.003	3.141	4.081	1.946	1.057	9.466
	70	5.926	15.86	4.293	7.179	8.619	3.147	4.463	4.332	3.274	4.607	2.312	1.127	10.692
	75	6.518	19.74	5.028	7.335	8.994	3.521	5.256	4.472	3.503	5.374	2.598	1.250	12.800

Supplementary Materials - Figures

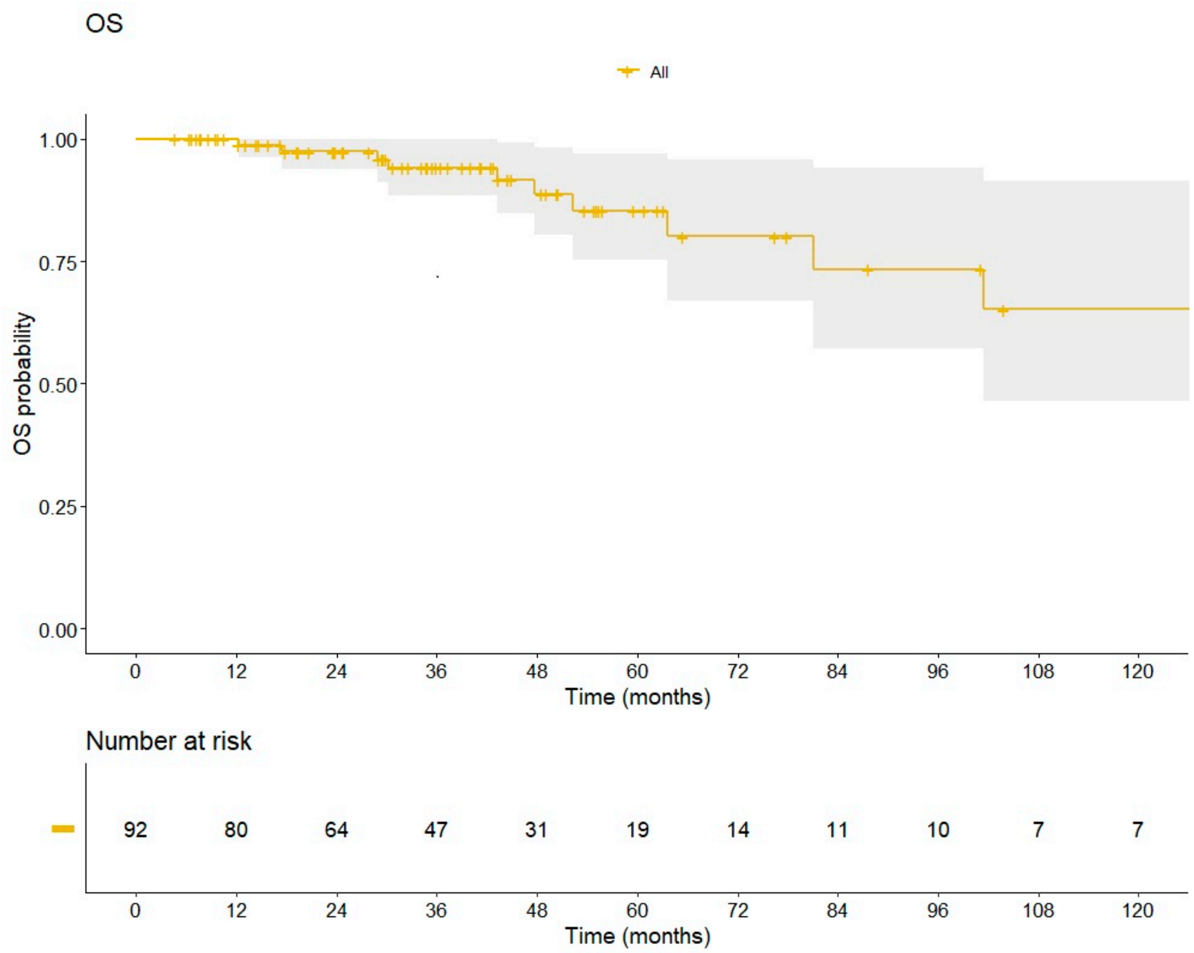

Figure S1. Overall Survival

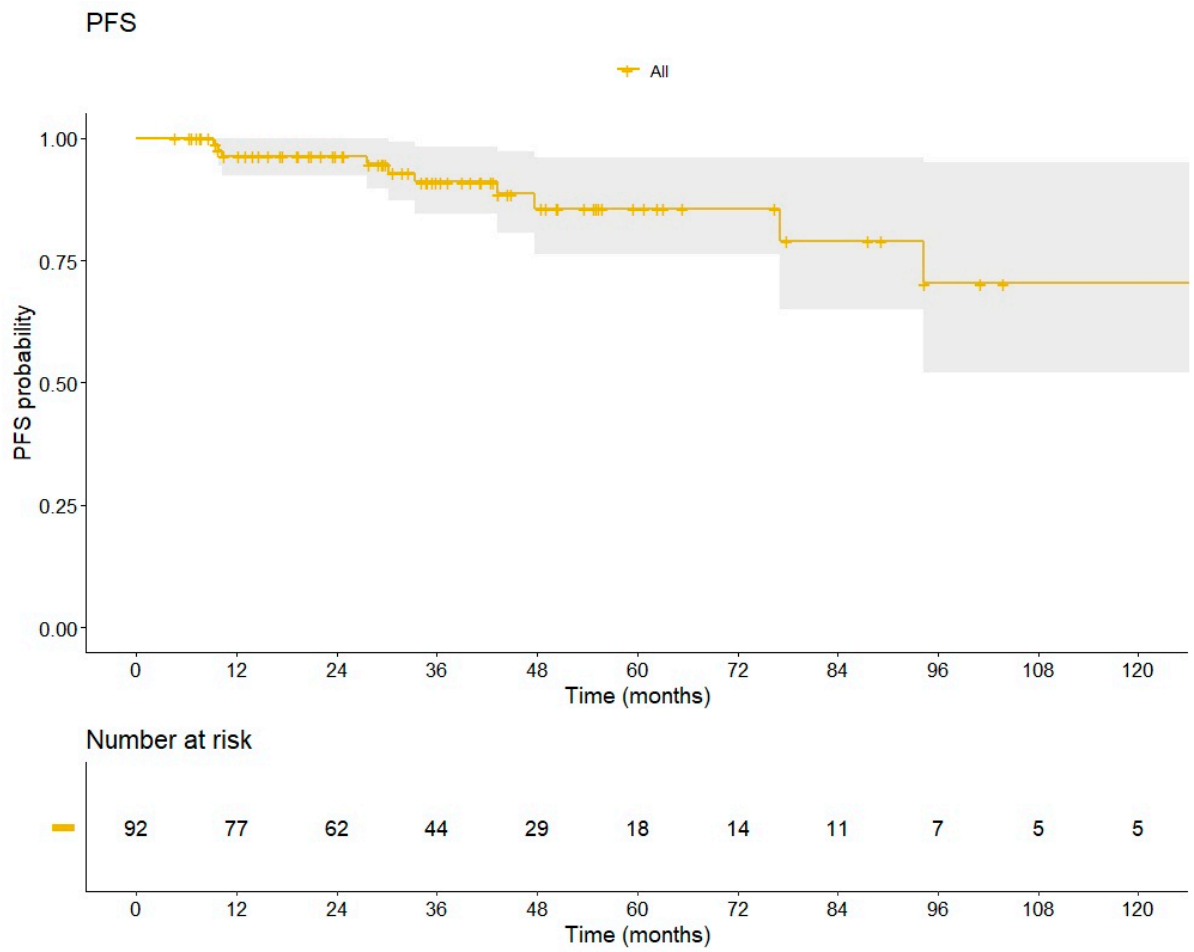

Figure S2. Progression-Free Survival (5)

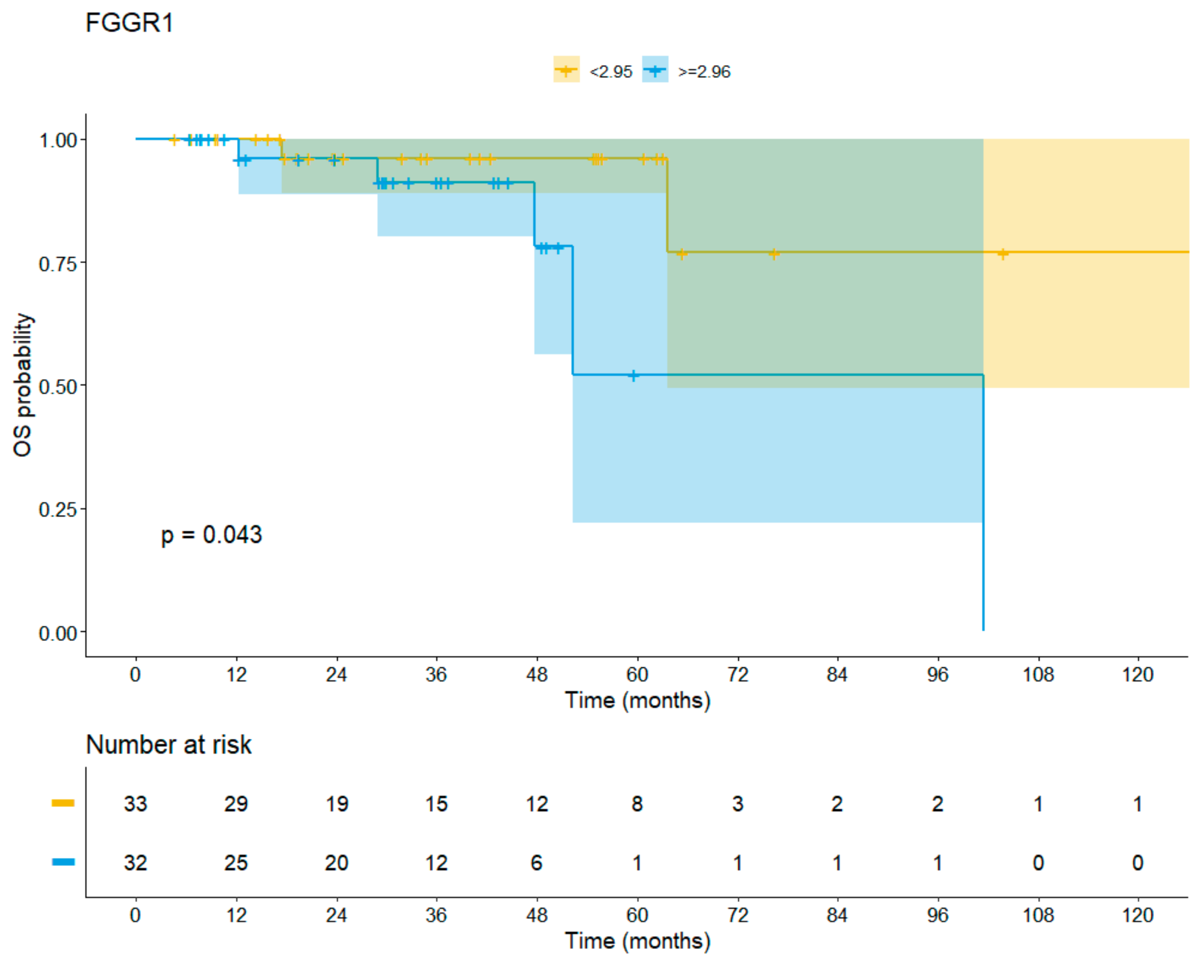

Figure S3. Association of *FGFR1* and Overall Survival

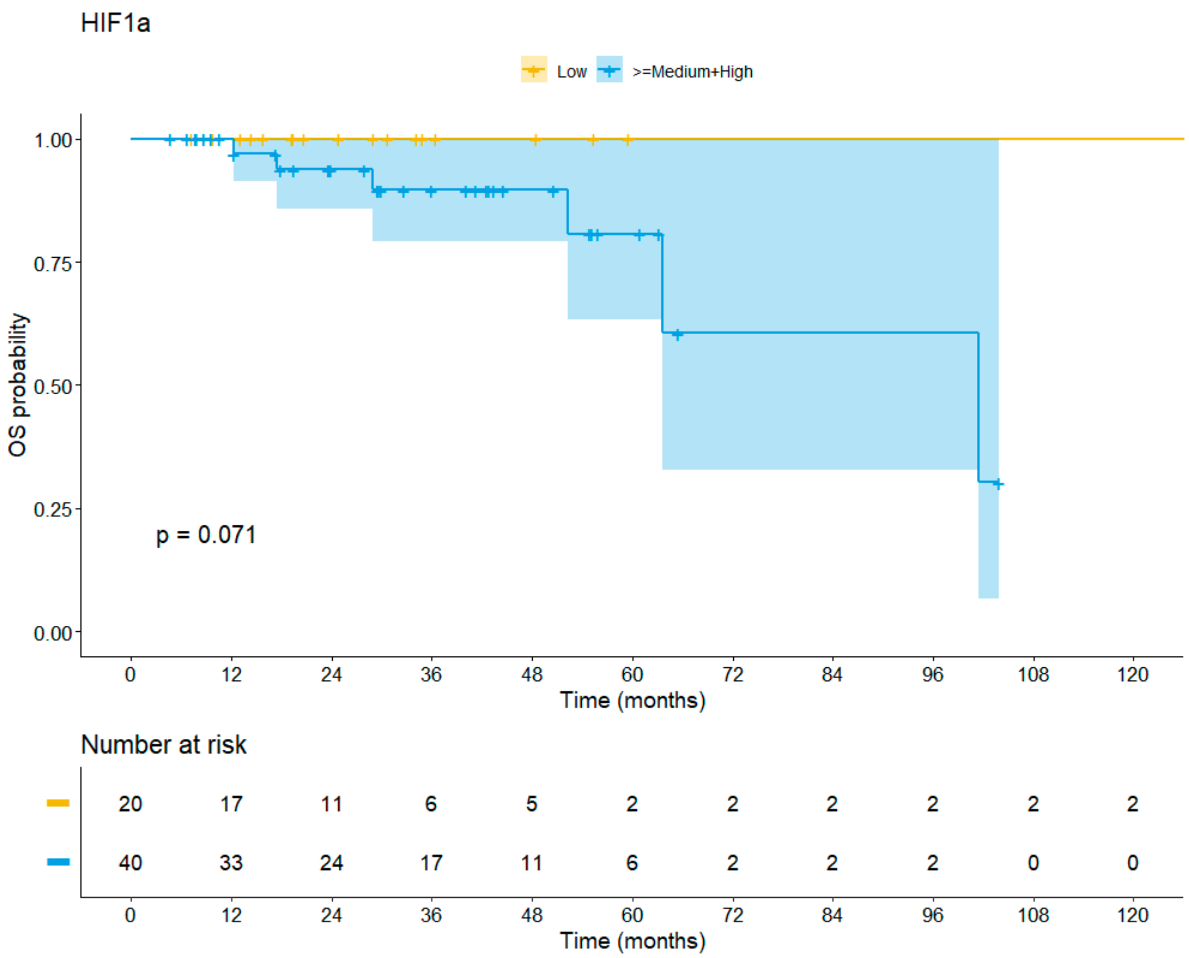

Figure S4. Association of *HIF1-alpha* and Overall Survival

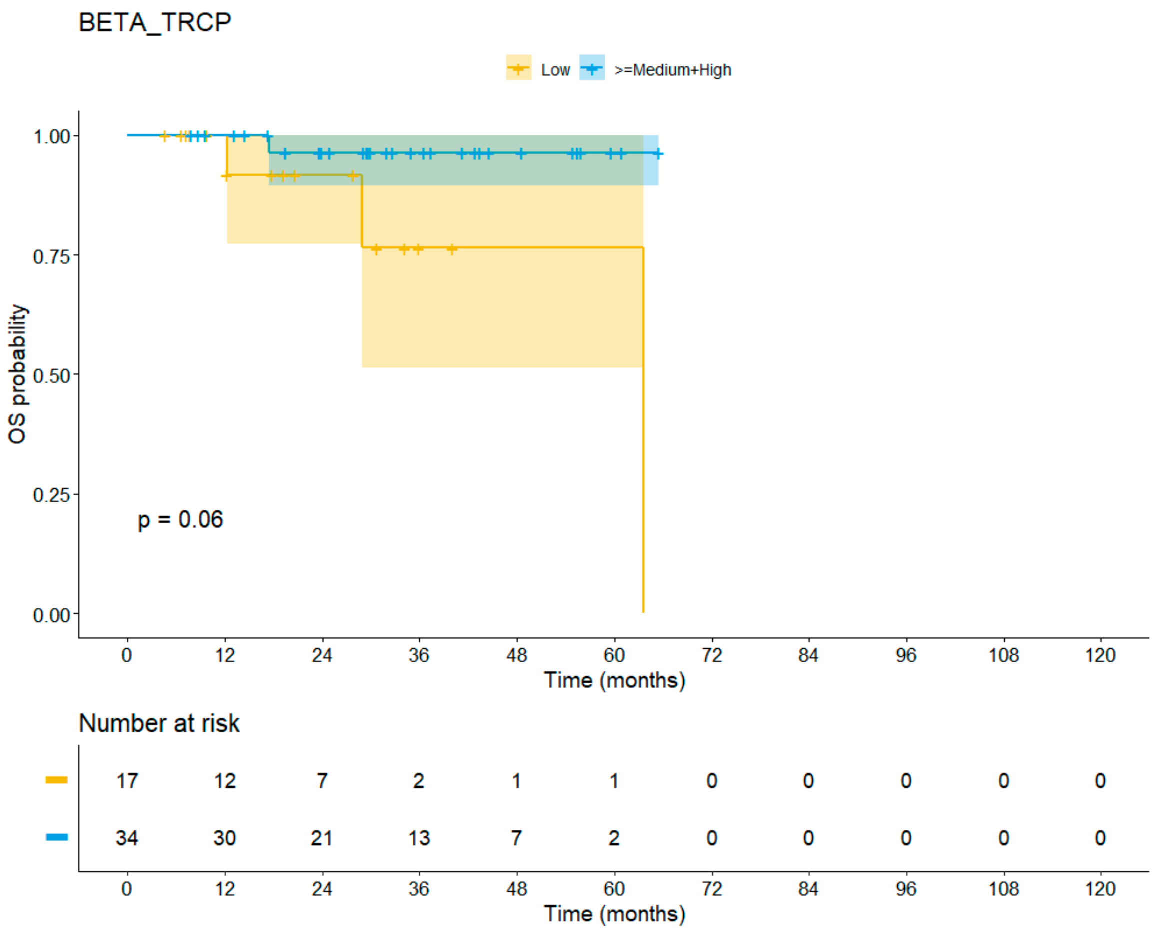

Figure S5. Association of *BTRCP* and Overall Survival

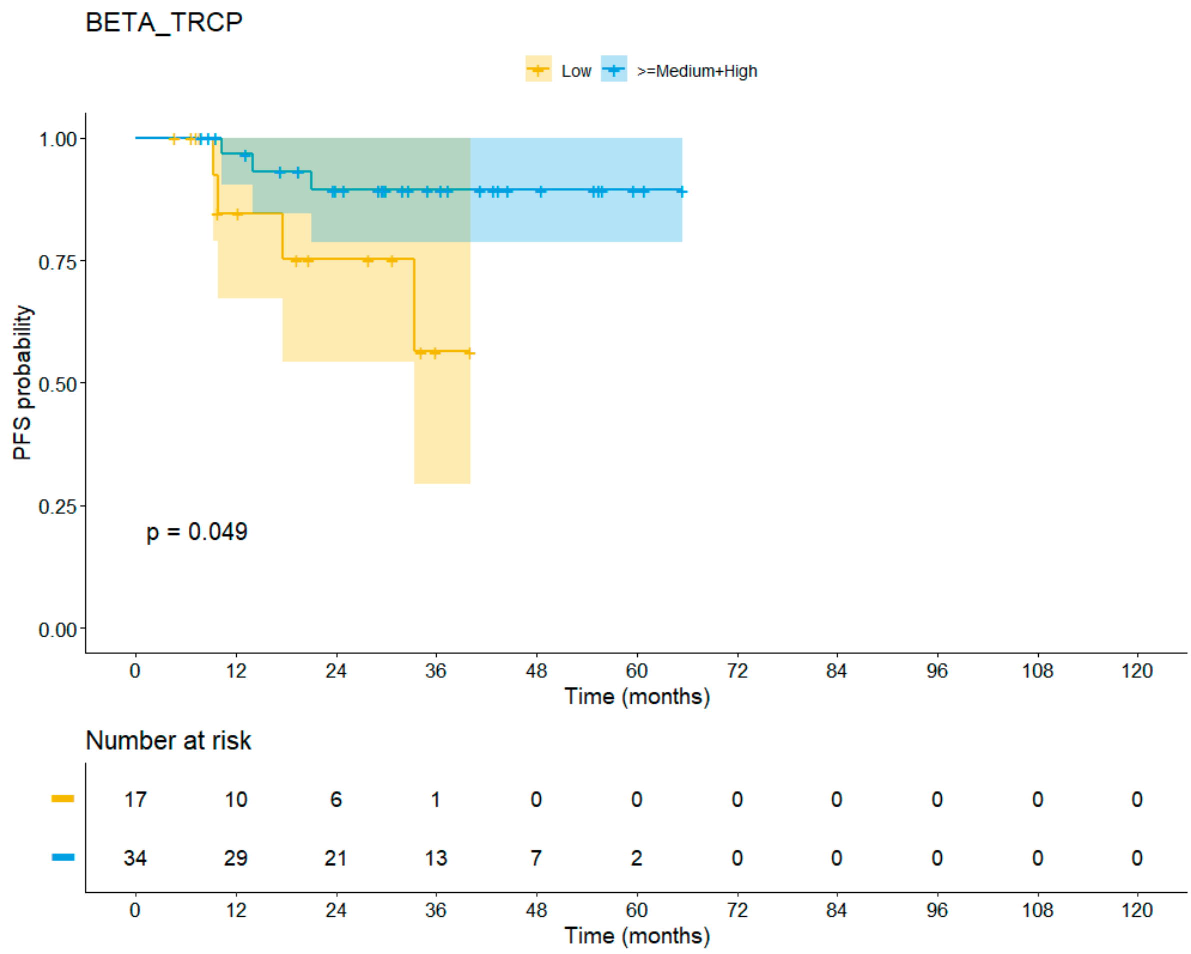

Figure S6. Association of *BTRCP* and Progression-Free Survival
